# Fungal Endophthalmitis Outbreak after Cataract Surgery, South Korea, 2020

**DOI:** 10.3201/eid2811.220361

**Published:** 2022-11

**Authors:** Soo Jeong Yoon, Soo Hyun Kim, Hyun Jung Bahk, Yeong Seo Ahn, Ji Joo Lee, Hye Jin Kim, Ha Jin Lim, Min Ji Choi, Jong Hee Shin, Yeon-Kyeng Lee

**Affiliations:** Korea Disease Control and Prevention Agency, Cheongju, South Korea (S.J. Yoon, H.J. Bahk, Y.S. Ahn, J.J. Lee, H.J. Kim, Y.-K. Lee);; Chonnam National University Medical School, Hwasun, South Korea (S.H. Kim, H.J. Lim, M.J. Choi, J.H. Shin)

**Keywords:** fungi, Fusarium, cataract, surgery, endophthalmitis, healthcare-associated infections, South Korea

## Abstract

In November 2020, an unusual increase in fungal endophthalmitis cases after cataract surgery was reported to the Korea Disease Control and Prevention Agency, South Korea. We initiated an outbreak investigation to identify the cause. We identified 156 cases nationwide, 62 confirmed and 94 probable. Most case-patients were exposed during surgery to ocular viscoelastic devices (OVDs) from the same manufacturer (company A). We isolated *Fusarium* spp. from 50 confirmed cases. Molecular identification of 39 fungal isolates from clinical samples and 13 isolates from OVDs confirmed *F. oxysporum* caused the infections. The risk ratio for fungal endophthalmitis from company A’s OVDs was 86.0 (95% CI 27.4–256.9), much higher than risk from other manufacturers’ products. We determined this fungal endophthalmitis outbreak was caused by a contaminated lot of OVDs and recommended discontinued use of this product. Early recognition of outbreaks and joint responses from related government agencies can reduce risk for fungal endophthalmitis.

Although the number of cataract surgery procedures is increasing globally because of an aging population, the incidence of postoperative endophthalmitis is declining because hygiene and surgical environments have improved (*1*,*2*). Postsurgical fungal endophthalmitis is difficult to diagnose because symptoms, such as decreased vision and eye pain, are nonspecific (*3*). Most cases of postoperative endophthalmitis are caused by bacteria, and ≈75% occur within 1 week after surgery (*4*). Because the symptoms of bacterial and fungal endophthalmitis are similar, intraocular fluid culture is crucial for an accurate diagnosis (*5*). Preventing serious complications such as vision loss requires immediate diagnosis, vitrectomy, and long-term antifungal therapy (*6*,*7*).

Postoperative endophthalmitis rarely occurs in South Korea; only ≈63 cases are reported per 100,000 cataract surgeries (*8*). However, the Korean Ophthalmological Society (KOS) recognized a sudden increase in endophthalmitis cases after cataract surgeries during September–November 2020, when ≈100 cases were reported nationwide. Cases included clinical findings of fungal endophthalmitis, including isolation of *Fusarium* species. Thus, in November 2020, KOS informed the Korea Disease Control Agency (KDCA), which promptly collaborated with the Korea Ministry of Food and Drug Safety (KMFDS) to investigate the unusual increase in fungal endophthalmitis, identify the cause, and recommend control measures. 

During the epidemiologic investigation, KMFDS collected commercially available samples of ocular viscoelastic devices (OVDs) from 6 manufacturers to conduct quality testing. OVDs are substances injected under the cornea to maintain the shape of the eye during cataract surgery and remain in the eyeball until the last step of surgery, when the OVD is removed. Thus, contaminated OVDs can cause intraocular infection. We describe an outbreak of fungal endophthalmitis after cataract surgery and confirmation of the cause through epidemiologic and microbiologic investigations.

## Materials and Methods

### Outbreak Determination

To determine whether the cases reported by KOS could be classified as an outbreak, KDCA analyzed data from Health Insurance Review and Assessment (HIRA) service records from the previous 3 years. Among 1,614,961 cataract surgeries performed during January 2018–September 2020, we estimated 702 (0.04%) cases of endophthalmitis, among which only 25 (0.002%) cases were presumed to be fungal infections. Thus, KDCA judged infections that occurred after September 2020 comprised an outbreak and suspected the cause was a contaminated batch of OVDs used in case-occurrence ophthalmology hospitals nationwide. 

### Endophthalmitis Case Investigation

KDCA documented reported endophthalmitis cases after cataract surgeries during September 1, 2020–January 11, 2021, in 101 referral centers nationwide. During that time, 182 cases were reported from certified tertiary hospitals, general hospitals (including ophthalmology departments), and specialized ophthalmology hospitals and in 45 referral centers. KDCA developed 2 epidemiologic investigation forms: 1 for hospitals that performed cataract surgery and 1 for referral centers that treated endophthalmitis. Ophthalmologists from hospitals and referral centers completed the epidemiologic investigation forms. Data collected from 69 hospitals where cataract surgeries were performed comprised patients’ demographic information, date of cataract surgery, date of endophthalmitis diagnosis, and the OVD brand and batch used in each surgery. Data collected from 45 referral centers comprised patient sample culture results and endophthalmitis treatment methods. In addition, the KDCA epidemiologic investigation team collected and reviewed medical records for all 182 cases from 45 referral centers.

### Case Definition

**Figure 1:**
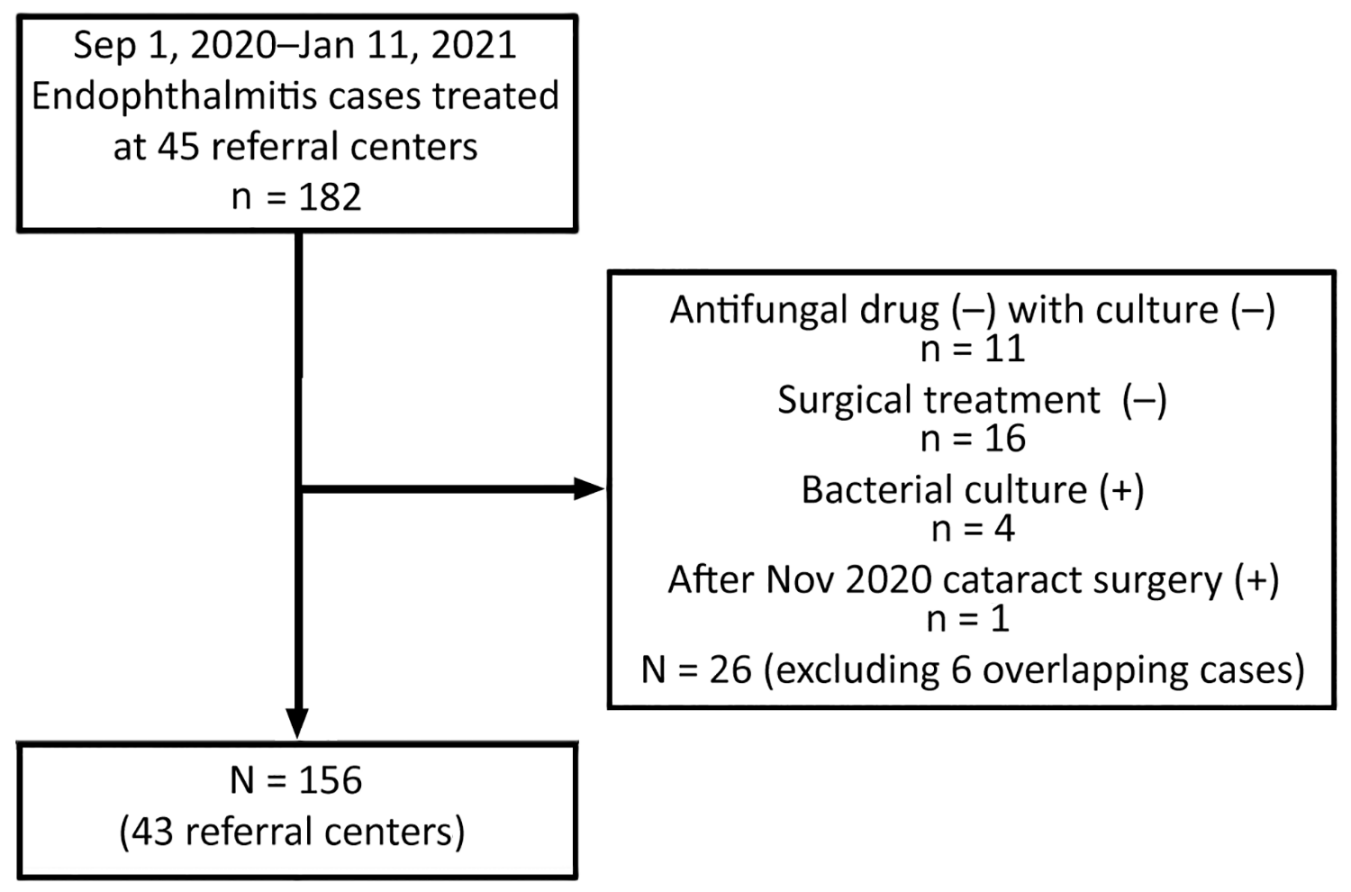
Flowchart for case-patient selection during fungal endophthalmitis outbreaks after cataract surgery, South Korea, 2020. Surgeries took place at 69 cataract surgery hospitals during September 1–November 30, 2020, and cases of *Fusarium oxysporum* endophthalmitis were identified during September 1, 2020–January 11, 2021.

For this outbreak, we defined a case as endophthalmitis in a patient who had cataract surgery during between September 1–November 30, 2020; had >1 of hypopyon, vitreous opacity, artificial lens infiltration, or retinal infiltration; had been transferred to an advanced ophthalmological hospital (retinal surgery hospital) nationwide; and had received antifungal drugs and surgical treatment under the advice of KOS. We defined a confirmed case as endophthalmitis in a patient in a referral center who had >1 positive fungal culture test results from surgical specimens, anterior chamber fluid, lens, or vitreous body. We defined a probable case as endophthalmitis in a patient who had negative fungal culture but who received antifungal treatment after a diagnosis of fungal endophthalmitis. We excluded patients whose samples tested positive for bacteria and those who had negative cultures and did not receive antifungal drugs. In all, the study encompassed 156 case-patients, 62 with confirmed cases and 94 with probable cases, from 43 referral centers nationwide (Figure 1).

### OVD Data Collection

We investigated the brands of OVD and other materials used in the 69 hospitals that performed cataract surgeries for the 156 identified case-patients. We used the 2020 HIRA Drug Supplier data records to identify the quantity of 6 brandstypes of OVDs supplied and when OVDs were sold to the hospitals.

### Laboratory Testing

KDCA designated a pathogen laboratory to conduct species identification and genotyping tests, including sequencing, to confirm whether isolates obtained from OVDs and patient samples were identical strains. KDCA sent 39 *Fusarium* spp. isolates collected from patients at 16 hospitals and 13 fungal isolates from OVDs that were collected by KMFDS and 2 university hospitals for sequencing. All 52 isolates were submitted for matrix-assisted laser desorption/ionization time-of-flight mass spectrometry analysis on VITEK MS (bioMérieux, https://www.biomerieux.com) or ASTA MicroIDSys (ASTA Inc, https://www.astams.co.kr). Sequencing identified the internal transcribed spacer and D1/D2 domain of 26S ribosomal DNA and *Fusarium*-specific translation elongation factor 1-alpha (*TEF1α*) gene. Sequencing analysis was performed by Macrogen (https://www.macrogen.com), and we used the BLAST (https://blast.ncbi.nlm.nih.gov/Blast.cgi) database to identify species.

We chose 12 clinical isolates for multilocus sequencing typing (MLST) analysis to confirm the genotype by using *TEF1α*, *RPB1*, and *RPB2* as target genes (*9*,*10*). We selected these isolates because they were collected from patients whose surgeries occurred during September–November 2020 in 6 certified tertiary hospitals evenly distributed throughout the nation. We also used isolates from OVDs collected by KMFDS and a university hospital. We analyzed genotypes of control strains for comparison with the outbreak strain. The control strains included 2 clinical isolates (1 from an eye specimen and the other from sputum) obtained before the outbreak period and 1 environmental isolate from the Korean Collection for Type Cultures (KCTC; https://kctc.kribb.re.kr), KCTC strain no. KCTC16654. We performed phylogenetic analysis by maximum-likelihood method using Kimura 2-parameter model and bootstrap analysis of 1,000 replications in MEGA version 11.0.11 (*11*). To elucidate the clustering of outbreak strains, we collected further outgroup data from GenBank.

### Data Analysis

We found that the monthly number of cataract surgeries performed in hospitals and the HIRA records for the monthly supply of OVDs were almost identical. Thus, we used the number of OVDs supplied to estimate the total number of cataract surgeries. We calculated the risk ratio and 95% CI by comparing the number of surgeries involving contaminated OVDs from 1 manufacturer, company A, and the occurrence of endophthalmitis with surgeries involving the other 5 OVD brands. We used EpiInfo (Centers for Diseases Control and Prevention, https://www.cdc.gov/epiinfo) for the statistical analysis.

## Results

### Epidemiologic Investigations

The mean age of the 156 case-patients was 66 years; 55 (35.3%) patients were in their 60s. Most (55%) patients had >1 underlying condition, including diabetes and high blood pressure. The left eye was affected in 65 (42%) cases; the right eye was affected in the remaining 91 cases (58%). More women (62%) than men were affected. The mean time from cataract surgery to the onset of symptoms was 24.3 days (range 1–84 days) (Table 1).

**Table 1:** Characteristics of patients with fungal endophthalmitis after cataract surgery, South Korea, 2020

Characteristics	No. (%), n = 156	Mean (+SD)
Sex		
M	59 (37.8)	
F	97 (62.2)	
Age range, y		66.3 (10.9)
<59	44 (28.2)	
60–69	55 (35.2)	
70–79	41 (26.3)	
>80	16 (10.3)	
Underlying conditions*		
None	70 (44.9)	
>1	86 (55.1)	
Involved eye		
Left	65 (41.7)	
Right	91 (58.3)	
Date of symptom onset		
October 2020	35 (22.4)	
November 2020	92 (59.0)	
December 2020	27 (17.3)	
January 1–11, 2021	2 (1.3)	
Latent period, d†		24.3 (14.8)
0–14	42 (26.9)	
15–28	60 (38.5)	
29–42	38 (24.4)	
43–56	11 (7.1)	
57–70	4 (2.6)	
70–84	1 (0.6)	

*Includes hypertension, diabetes mellitus, heart disease, cerebrovascular disease, hyperlipidemia, and other conditions. †Time from surgery to clinical manifestations.

**Figure 2:**
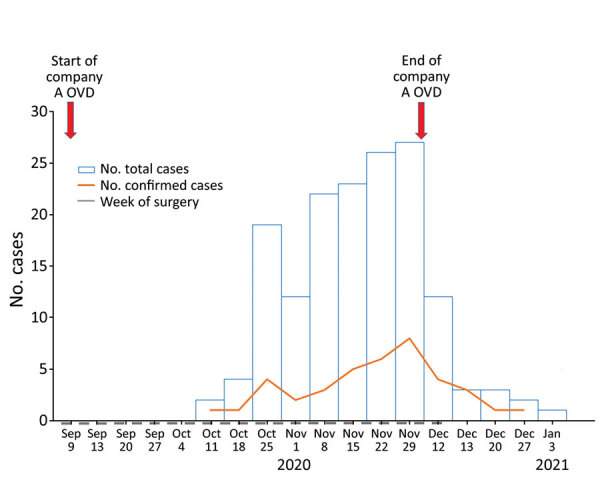
Epidemic curve of fungal endophthalmitis outbreaks after cataract surgery, South Korea, 2020. The curve shows 156 cases of fungal endophthalmitis after cataract surgery. Cases were linked to ophthalmic viscoelastic devices from company A (A-OVD) contaminated with *Fusarium oxysporum*.

A batch of fungal-contaminated OVDs was supplied to medical institutions nationwide by 1 manufacturer, company A, in September 2020. Ophthalmology departments stopped using this product after KOS recognized the outbreak and contacted KDCA in November 2020. Correspondingly, the number of cases of fungal endophthalmitis occurring within 3 months of cataract surgery increased after September and then decreased again after November; 35 cases were reported in October, 92 in November, 27 in December, and 2 in January, the last on January 11, 2021 (Figure 2).

**Figure 3:**
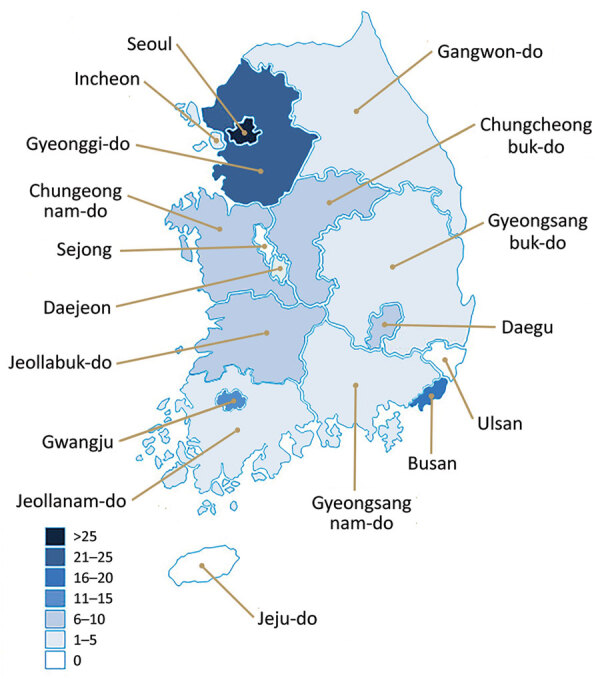
Geographic distribution of cases in fungal endophthalmitis outbreak after cataract surgery, South Korea, 2020. Surgeries took place during September 1–November 30, 2020, and cases of *Fusarium oxysporum* endophthalmitis were identified during September 1, 2020–January 11, 2021.

Fungal endophthalmitis was reported from 14 cities and provinces nationwide; no cases were reported for Sejong, Ulsan, or Jeju (Figure 3). In 152 (98%) cases, OVDs from company A were used in cataract surgery. Cases were reported from 65 of the 69 medical institutions in the study, which comprised 60 clinics, 8 specialized ophthalmology hospitals, and 1 general hospital (Table 2).

**Table 2:** Ophthalmic viscoelastic device brands and microbiological spectrum of 156 cases of fungal endophthalmitis after cataract surgery, South Korea, 2020*

Brand of OVD	No. (%) cases	*Fusarium* spp.	Other fungus†	Culture-negative	Unknown
A	152 (97.4)	50	11	90	1
B	2 (1.4)	NA	1	1	NA	
C	1 (0.6)	NA	NA	1	NA	
D	1 (0.6)	NA	NA	1	NA	
E	0	NA	NA	NA	NA	
F	0	NA	NA	NA	NA	
Total	156 (100)	50	12	93	1

*OVD, ophthalmic viscoelastic device; NA, not applicable. †Includes *Aspergillus* (n = 6), *Acremonium* (n = 4), and *Exophiala spp*. (n = 1), and undetermined type (n = 1).

### Microbiologic Results

We isolated *Fusarium* species from 50 (33%) patient samples, and identified other fungal species in 12 cases, including *Aspergillus* in 6 cases, *Acremonium* in 4 cases, *Exophiala* in 1 case, and an undetermined type in 1 case. In addition, 93 cases had negative cultures and 1 case had an unclear microbial test result (Table 2).

KMFDS determined 2 strains of fungus contaminated a batch of OVDs from company A. We collected 39 *Fusarium* isolates from case-patients and all isolates were confirmed as *F. oxysporum* by visual and microscopic fungal identification tests and matrix-assisted laser desorption/ionization time-of-flight mass spectrometry in the pathogen laboratory.

**Figure 4:**
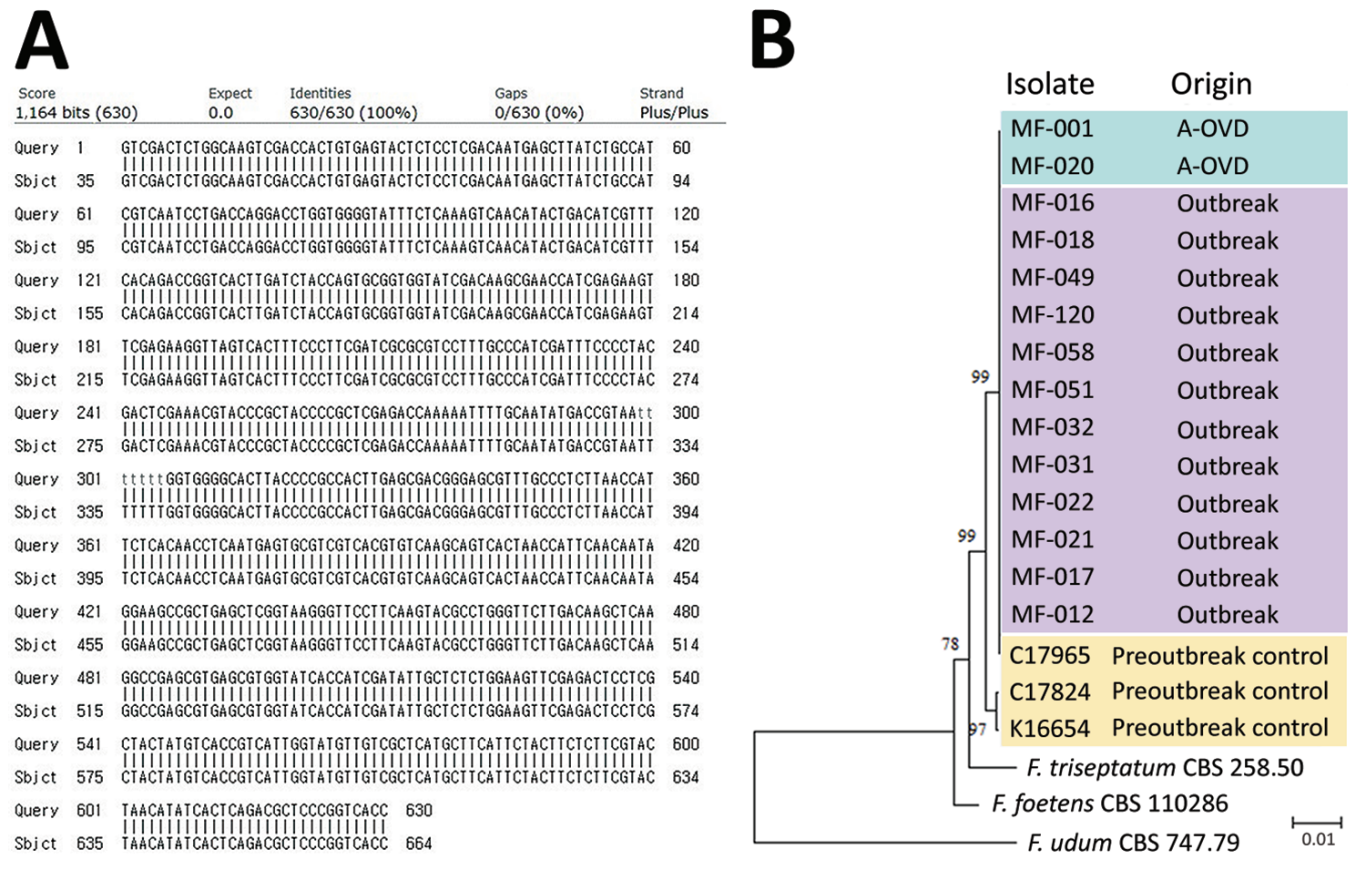
Sequence of outbreak strain matched to *Fusarium oxysporum* strain in a study of fungal endophthalmitis outbreaks after cataract surgery, South Korea, 2020. A) We matched 2020ASF-167 translation elongation factor 1-α (*TEF1α*) gene of the outbreak strain with a control strain (GenBank accession no. MN646762.1) B) Phylogenetic tree of the concatenated sequences from 3 genes (*TEF1α*, *RPB1*, and *RPB2*). Phylogenetic data include outbreak strain from 2 A-OVDs, 12 clinical isolates, 3 preoutbreak controls, and 3 outgroup sequences collected from GenBank database. Scale bar indicates the number of nucleotide substitutions per site. A-OVD, ocular viscoelastic devices from company A.

Sequencing typing of *TEF1α*, a *Fusarium*-specific target gene, confirmed that the *TEF1α* sequence of the *F. oxysporum* strain registered in the database matched 100% with the submitted query of 630 bp (Figure 4, panel A). MLST revealed that 12 *F. oxysporum* isolates from patients and 2 isolates from the contaminated OVDs were same type, which we classified as clade A. Of 3 control strains, 2 strains (1 clinical strain from an eye specimen obtained before the outbreak period and environmental strain no. KCTC16654 from KCTC) disclosed different MLST types, but the clinical strain from sputum showed the same clade A type (Figure 4, panel B).

We conducted an onsite epidemiologic investigation on December 21, 2020, at an ophthalmologic clinic where cases of postsurgery endophthalmitis had occurred in October 2020. We collected 13 types of environmental samples from materials and devices used for surgeries, from water tap, and from a refrigerator in the operating room. We cultured the environmental samples and found no discernable microbes, including fungi.

### Risk Assessment

We used ophthalmic surgery supply records from HIRA for the 6 brands of OVDs to estimate the number of cataract surgeries that occurred during September–November 2020. We assumed that most OVDs were used for cataract surgery and that 1 OVD was used per surgery. Then, using patients’ medical records and case-study reports, we determined 62 confirmed cases and 94 probable cases of fungal endophthalmitis after cataract surgery occurred during the study period. We excluded 1 case from our statistical analysis because it had insufficient information regarding the OVD supplied. We calculated the rate of infection for company A OVDs compared with the 5 other OVD brands. We found the incidence risk for infection for manufacturer A OVDs was 0.3% and risk for infection from the other 5 OVDs was 0.004%, indicating an 86-fold higher risk for fungal endophthalmitis when manufacturer A OVDs were used compared with the other brands (Table 3).

**Table 3:** Relative risk for fungal endophthalmitis after cataract surgery using ophthalmic viscoelastic device from company A, South Korea, 2020*

Brand of OVD	No. cases†	No. surgeries	Incidence risk per 100,000 population	Risk ratio (95% CI)
Company A OVD	152	49,193	309.0	86.0 (27.4–269.7)
Other OVDs, referent	3	83,554	3.6	

*OVD, ophthalmic viscoelastic device. †Among 156 total cases; excludes 1 case that used an OVD without sufficient database information.

### Public Health and Regulatory Actions

On November 23, 2020, as the epidemiologic investigation began, KDCA recommended that KOS immediately issue a warning to its surgeons to stop using the suspected devices. Once fungal contamination was confirmed in a batch of OVDs from company A, KMFDS implemented measures to stop production and recall the product on December 11, 2020.

## Discussion

In this investigation of an outbreak of endophthalmitis after cataract surgeries, we identified OVDs as a common substance used in nearly all the surgical procedures that led to endophthalmitis and confirmed fungal contamination in a batch of product from 1 manufacturer. Using genetic sequencing, we identified *F. oxysporum* as the causative fungus in patient samples and isolates from contaminated OVDs. 

In 2005, a total of 20 cases of postoperative fungal endophthalmitis infection caused by contaminated OVDs were reported in the Czech Republic (*12*). Similarly, 47 cases of infection caused by intraocular dye and contaminated medication from the same manufacturer were reported in the United States (*13*). In addition, retrospective studies analyzing the causes, treatments, and prognoses on the basis of hospital medical records over several years and a report on treatment results in 7 individual cases have been published (*5*,*14*,*15*). In 2 previous outbreaks involving contaminated medical products in the United States, epidemiologic investigations of the reported medical institutions identified intraocular dye used in retinal surgery that was contaminated with *F. incarmatum-equiseti* (*13*) and prefilled saline flush syringes contaminated with *Burkholderia cepacia* complex because of an inappropriate sterilization process (*16*). In both outbreaks, subsequent investigations expanded nationwide and documented additional cases (*13*,*16*). In this outbreak, we investigated postoperative infections reported across South Korea and used data to trace cases to specific cataract surgery hospitals because no information was available on the specific medical institutions where the cases occurred and South Korea does not have a reporting system for case data, as the United States does.

Most (70%–95%) post–cataract surgery endophthalmitis cases involve gram-positive bacteria (*17*); fungal causes are quite rare, accounting for <5%. Causative fungi are usually *Aspergillus*, *Candida*, *Acremonium*, and *Fusarium* spp. (*5*,*7*). *F. oxysporum*, the causative agent in the outbreak we report, occurs in plants and soil and secretes mycotoxins that can cause local and systemic infections in humans (*12*). Microbiologic testing identified *Fusarium* spp. in ≈30% of 156 patient samples obtained. Because of drugs administered during treatment and technical problems during testing, microbiologic testing identifies the causative agent in only 30%–50% of endophthalmitis cases (*12*,*18*). Therefore, we did not rule out *Fusarium* spp. infection in patients whose samples tested positive for other fungi or tested negative altogether. 

This outbreak occurred at various clinics, specialized ophthalmology hospitals, and general hospitals across the country over a short period; thus, we inferred that the possibility of infection from the environment was small. The incubation period for bacterial endophthalmitis is relatively short, just 7 days (*17*). For cases in our outbreak, patient symptoms were delayed until ≈24 days after surgery, which is a clinical manifestation of fungal endophthalmitis (*5*); moreover, fungi were cultured in samples obtained from the eye fluid of some patients. Therefore, we hypothesized that the most likely cause of the outbreak was contamination of the eyeball during surgery with a fungus emanating from a common source. After the batch of contaminated OVDs from company A were supplied for to institutions for use, the number of reported fungal endophthalmitis infections rapidly increased, but after the cause of the outbreak was eliminated, infections decreased. Because the data collection period was prolonged, we examined the suspected causative agent by confirming that the same fungus was in the suspect OVDs and patient samples before we conducted the final data analysis. The purpose of the epidemiologic investigation was to identify the causative agent for the outbreak; therefore, we did not conduct a case–control study to determine any other potential causes. We did not have epidemiologic bias when calculating the risk ratio because we equally applied the conditions, estimated number of cataract surgeries, and parameters for case inclusion to contaminated OVDs and other OVDs.

The first limitation of this study is that we excluded patients who were not sent to a higher-level hospital and had not undergone surgical treatment; thus, we might not have identified the actual number of infected persons. Second, we did not investigate possible causes of fungal endophthalmitis other than the OVDs. Third, we did not investigate other patients whose surgery involved the contaminated product but who did not seek care for fungal infection. However, this study reports a large-scale fungal endophthalmitis outbreak related to the use of OVDs. In this case, fungal-contaminated devices were supplied nationwide beginning in September 2020 and used for surgical procedures in many facilities. The outbreak was recognized at the end of November 2020, by which time >100 cases had occurred. We surmise delayed outbreak recognition was mainly due to the ambiguous symptoms and slow progression of fungal endophthalmitis, in addition to difficulties in performing fungal culture. A further follow-up study of infected patients is needed to determine their prognosis and to investigate possible factors other than contaminated OVDs that could cause fungal endophthalmitis. Such a study also could collect samples from cataract surgery patients for whom similarly contaminated OVDs were used but who did not develop infection.

In summary, we identified *F. oxysporum* as the cause of an outbreak of fungal endophthalmitis after cataract surgery in South Korea and confirmed the association between the causative agent and the outbreak through an epidemiologic investigation. We concluded that a batch of OVDs, devices commonly used at ophthalmic hospitals nationwide, was contaminated with *F. oxysporum*, causing post–cataract surgery fungal endophthalmitis throughout the country. We identified the same *F. oxysporum* strain in contaminated OVDs and in patient samples, and most cases occurred among patients whose OVDs came from 1 manufacturer. The data we provide can help estimate infection factors, provide early recognition of simultaneous outbreaks, and aid rapid quarantine of suspected causative agents in similar cases in the future.

## References

[1] Keay L, Gower EW, Cassard SD, Tielsch JM, Schein OD. Postcataract surgery endophthalmitis in the United States: analysis of the complete 2003 to 2004 Medicare database of cataract surgeries. Ophthalmology. 2012;119:914–22. 10.1016/j.ophtha.2011.11.023 22297029 PMC3343208

[2] Nowak MS, Grzybowski A, Michalska-Małecka K, Szaflik JP, Kozioł M, Niemczyk W, et al. Incidence and characteristics of endophthalmitis after cataract surgery in Poland, during 2010–2015. Int J Environ Res Public Health. 2019;16:2188. 10.3390/ijerph16122188 31226859 PMC6617312

[3] Rahmani S, Eliott D. Postoperative endophthalmitis: a review of risk factors, prophylaxis, incidence, microbiology, treatment, and outcomes. Semin Ophthalmol. 2018;33:95–101. 10.1080/08820538.2017.1353826 29172849

[4] Endophthalmitis Vitrectomy Study Group. Results of the Endophthalmitis Vitrectomy Study. A randomized trial of immediate vitrectomy and of intravenous antibiotics for the treatment of postoperative bacterial endophthalmitis. Arch Ophthalmol. 1995;113:1479–96. 10.1001/archopht.1995.01100120009001 7487614

[5] Narang S, Gupta A, Gupta V, Dogra MR, Ram J, Pandav SS, et al. Fungal endophthalmitis following cataract surgery: clinical presentation, microbiological spectrum, and outcome. Am J Ophthalmol. 2001;132:609–17. 10.1016/S0002-9394(01)01180-1 11704021

[6] Chen YH, Chen JT, Tai MC, Chou YC, Chen CL. Acute postcataract endophthalmitis at a referral center in northern Taiwan: Causative organisms, clinical features, and visual acuity outcomes after treatment: A retrospective cohort study. Medicine (Baltimore). 2017;96:e8941. 10.1097/MD.0000000000008941 29245262 PMC5728877

[7] Jeong SH, Cho HJ, Kim HS, Han JI, Lee DW, Kim CG, et al. Acute endophthalmitis after cataract surgery: 164 consecutive cases treated at a referral center in South Korea. Eye (Lond). 2017;31:1456–62. 10.1038/eye.2017.85 28548647 PMC5639198

[8] Kim SH, Yu MH, Lee JH, Kim SW, Rah SH. Endophthalmitis after cataract surgery in Korea: a nationwide study evaluating incidence and risk factors in a Korean population. Yonsei Med J. 2019;60:467–73. 10.3349/ymj.2019.60.5.467 31016909 PMC6479122

[9] Moretti ML, Busso-Lopes AF, Tararam CA, Moraes R, Muraosa Y, Mikami Y, et al. Airborne transmission of invasive fusariosis in patients with hematologic malignancies. PLoS One. 2018;13:e0196426. 10.1371/journal.pone.0196426 29698435 PMC5919535

[10] O’Donnell K, Gueidan C, Sink S, Johnston PR, Crous PW, Glenn A, et al. A two-locus DNA sequence database for typing plant and human pathogens within the *Fusarium oxysporum* species complex. Fungal Genet Biol. 2009;46:936–48. 10.1016/j.fgb.2009.08.006 19715767

[11] Kumar S, Tamura K, Nei M. MEGA: Molecular Evolutionary Genetics Analysis software for microcomputers. Comput Appl Biosci. 1994;10:189–91. 10.1093/bioinformatics/10.2.189 8019868

[12] Buchta V, Feuermannová A, Váša M, Bašková L, Kutová R, Kubátová A, et al. Outbreak of fungal endophthalmitis due to *Fusarium oxysporum* following cataract surgery. Mycopathologia. 2014;177:115–21. 10.1007/s11046-013-9721-5 24381050

[13] Mikosz CA, Smith RM, Kim M, Tyson C, Lee EH, Adams E, et al.; Fungal Endophthalmitis Outbreak Response Team. Fungal endophthalmitis associated with compounded products. Emerg Infect Dis. 2014;20:248–56. 10.3201/eid2002.131257 24447640 PMC3901475

[14] Alagöz N. Ten years after an outbreak of *Fusarium* endophthalmitis following cataract surgery. Arq Bras Oftalmol. 2020;83:454–6.3308482510.5935/0004-2749.20200106

[15] Yang YS. Results of extensive surgical treatment of seven consecutive cases of postoperative fungal endophthalmitis. Korean J Ophthalmol. 2009;23:159–63. 10.3341/kjo.2009.23.3.159 19794941 PMC2739967

[16] Brooks RB, Mitchell PK, Miller JR, Vasquez AM, Havlicek J, Lee H, et al.; Burkholderia cepacia Workgroup. Burkholderia cepacia Workgroup. Multistate outbreak of *Burkholderia cepacia* complex bloodstream infections after exposure to contaminated saline flush syringes: United States, 2016–2017. Clin Infect Dis. 2019;69:445–9. 10.1093/cid/ciy910 30346502 PMC6476681

[17] Durand ML. Bacterial and fungal endophthalmitis. Clin Microbiol Rev. 2017;30:597–613. 10.1128/CMR.00113-16 28356323 PMC5475221

[18] Choi SC, Cho HJ, Kim HS, Han JI, Lee DW, Cho SW, et al. Analysis of referred 113 patients with endophthalmitis after cataract surgery and associated prognostic factors. J Korean Ophthalmol Soc. 2016;57:420–8. 10.3341/jkos.2016.57.3.420

